# Expression of claudin-11, -23 in different gastric tissues and its relationship with the risk and prognosis of gastric cancer

**DOI:** 10.1371/journal.pone.0174476

**Published:** 2017-03-28

**Authors:** Youzhu Lu, Jingjing Jing, Liping Sun, Yuehua Gong, Moye Chen, Zeyang Wang, Mingjun Sun, Yuan Yuan

**Affiliations:** 1 Digestive department, The First Affiliated Hospital of China Medical University, Liaoning, Shenyang, Liaoning, China; 2 Tumor Etiology and Screening Department of Cancer Institute and General Surgery, The First Affiliated Hospital of China Medical University, Key Laboratory of Cancer Etiology and Prevention (China Medical University), Liaoning Provincial Education Department, Shenyang, Liaoning, China; National Cancer Center, JAPAN

## Abstract

Claudins play an important role in regulating the permeability of epithelial and endothelial cells and in the maintenance of cell polarity. We aimed to investigate expression of claudin-11, -23 in different gastric tissues and its relationship with clinicopathologic parameters and prognosis of gastric cancer. We compared their expression levels in the paired cancerous tissues versus those in the adjacent noncancerous tissues by real-time PCR, western blotting and immunohistochemistry. The results showed that the expression of claudin-11, -23 was greatly increased in paracancerous gastric tissue compared with cancerous tissue. We also compared their expression levels of tissues from gastric cancer, superficial gastritis, and atrophic gastritis by immunohistochemistry. The results indicated that the expression of claudin-11 and 23 was significantly higher in superficial gastritis than that in atrophic gastritis and gastric cancer. The expression of claudin-23 was significantly lower in atrophic gastritis than that in gastric cancer, but no obviously difference was observed for claudin-11. As for analysis of clinicopathologic parameters of gastric cancer, logistic multiple regression indicated that claudin-11 was significantly associated with sex, smoking, alcohol, *H*. *pylori* infection and Borrmann classification while claudin-23 was significantly associated with vessel cancer embolus. Cox multivariate survival analysis indicated that gastric cancer patients with negative claudin-23 expression had significantly longer overall survival. In conclusion, the expression of claudin-11, -23 was remarkably downregulated in gastric cancer. Abnormal expression of these proteins was significantly correlated with some clinicopathologic parameters. In particular, claudin-23 positive expression was associated with poor prognostic outcomes of gastric cancer patients and may therefore serve as an independent prognosticator of patient survival.

## Introduction

The gastric mucosal barrier is an important defensive mechanism that can protect gastric mucosa and prevent the occurrence of gastric diseases. The integrity of its structure and function not only prohibits diffusion of H+ from the gastric cavity to gastric mucosa and of Na+ from mucosa to the gastric cavity, but also defends against the invasion of harmful factors. The gastric mucosal barrier is mainly composed of epithelial cells and intracellular junctions. The tight junction, a multicomplex of membrane proteins, is the most essential component of the intracellular junction structure. Tight junction transmembrane proteins known as claudins, a family of 27 proteins, play a critical role in the maintenance of cell polarity and barrier function and in the permeability of epithelial cells [1–3]. Abnormal expression and distribution of claudin proteins may disrupt the structure and function of tight junctions, leading to damage of the gastric mucosal barrier and thereby allowing abnormal lateral diffusion of intracellular molecules and the invasion of bacteria and virulence factors from gastric mucosa into the organic body, which may result in the occurrence of many kinds of gastric diseases and even gastric cancer [4]. For example, claudin-1, which is highly expressed in the intestinal type of gastric cancer, is correlated with tumor invasion and migration and poor prognosis and overexpression of claudin-1 may promote the proliferation and migration of gastric cancer cells [5–7]. Downregulation of claudin-18 has been shown to be related to proliferation and migration of gastric cancer cells [8]. Moreover, some studies suggest that abnormal changes in claudin proteins may lead to the invasion, metastasis, and recurrence of gastric tumor, irrespective of early or late stage [9–11]. Therefore, abnormal expression of claudins may be closely related to the occurrence, progression, and prognosis of gastric cancer.

Claudin-11, -23 are another two important members of the claudin family. There is accumulating evidence that abnormal expression of these two proteins can disrupt the structure and function of tight junctions, leading to destruction of the barrier function of epithelial cells. Previous studies found that claudin-11, -23 are expressed abnormally in several malignant tumors. For example, in hepatocellular carcinoma and gallbladder carcinoma, downregulation of claudin-11 can promote the metastasis of tumors [12, 13]. Decreased expression of claudin-23 was observed in colonic cancer, and its low expression was reported to be related to the invasion of pancreatic carcinoma [14, 15]. However, there are few reports about the expression of claudin-11, -23 in gastric cancer, and existing studies have yielded conflicting results. For example, some studies showed that claudin-11 was upregulated in gastric cancer [16], whereas Agarwal et al. [17] found that it was downregulated in gastric cancer and further suggested that its downregulation in gastric epithelial cells may promote the invasion of cells. Decreased mRNA expression of claudin-23 has been observed in intestinal type of gastric cancer [18], but its protein expression level in gastric cancer is unclear. In addition, the relationship between the expression of claudin-11, -23 and the biologic behavior and prognosis of gastric cancer remains elusive. Therefore, whether claudin-11, -23 participate in the occurrence and progression of gastric cancer and their relationship with the prognosis of patients with gastric cancer should be further investigated.

In this study, we compared the expression of claudin-11, -23 in gastric cancer, atrophic gastritis, and superficial gastritis. We further investigated the relationship between the expression of claudin-11, -23 and the clinicopathologic parameters and prognosis of gastric cancer to elucidate the potential roles of claudin-11, -23 in the occurrence, progression, and prognosis of gastric cancer.

## Materials and methods

### Research subjects

A total of 218 patients with superficial gastritis and atrophic gastritis were selected from a health check-up program involving gastroscopy for gastric cancer screening in Zhuanghe, Liaoning Province, between 2008 and 2011. In addition, 109 patients with gastric cancer (of whom 93 had paracancerous atrophic gastritis and 109 had paracancerous superficial gastritis) who underwent surgical treatment without chemoradiotherapy or other therapy before surgery between 2012 and 2015 were enrolled from the anorectal department of the First Affiliated Hospital of China Medical University. All diagnoses were histologically determined based on the updated Sydney System for gastritis and the World Health Organization (WHO) criteria for gastric cancer [19], and tumors were staged using the 7th edition of the TNM staging system [20]of the International Union Against Cancer (UICC)/American Joint Committee on Cancer (AJCC) 2010 [21] on the basis of a postoperative pathologic examination. A 5-ml fasting venous blood sample was obtained for measurement for *H*. *pylori* serology. Data regarding sex, age, smoking, and alcohol consumption were obtained by questionnaire. Clinical characteristics and biomarkers of cancer patients were extracted from their medical record, including phase of progression, Borrmann classification, Lauren’s classification, TNM stage, vessel cancer embolus, tumor size, perineural invasion, family history and in situ expression of carcinoembryonic antigen (CEA) and glutathione S-transferase P1 (GSTP1). The follow-up inquiry was finished in July 2016, and complete prognostic information was obtained from 97 of the 109 patients with gastric cancer. There were no statistical differences among the different groups in terms of age and gender composition (P = 0.364; 0.840) (Table 1).

**Table 1:** Baseline information of the SG, AG and GC.

Variable	Total	Categories			*P*
		SG	AG	GC	
**Total n**	327	109	109	109	
**Age (years)**					
<60	186	68	59	59	0.364
≥60	141	41	50	50	
**Sex**					
Male	212	69	70	73	0.840
Female	115	40	39	36	
**Smoking**					
Yes	99	35	28	36	0.324
No	222	74	81	67	
**Alcohol**					
Yes	70	23	29	18	0.268
No	251	86	80	85	
***H*. *pylori* IgG**					
Seronegative	155	84	42	29	3.66*10^**−12**^
Seropositive	159	25	67	67	
**Family history**					
Yes	23			23	
No	79			79	

GC, gastric cancer. AG, atrophic gastritis. SG, superficial gastritis.

### Ethics statement

This study was approved by the Ethics Committee of the First Affiliated Hospital of China Medical University Shenyang, China, and written informed consent was obtained from all participants.

### RNA extraction and real-time PCR

Total tissue RNA was extracted with Trizol buffer (Thermo Fisher Scientific, Massachusetts, USA). RNA was reverse transcribed to cDNA using PrimeScript RT Master Mix (Takara). Real-time PCR was carried out on Eppendorf equipment using SYBR Premix Ex Taq (Takara, Liaoning, China) in accordance with the manufacturer’s protocols. The results were analyzed by the 2^-Δ^Ct method. The primer sequences for claudin-11, claudin-23, and glyceraldehyde 3 phosphate dehydrogenase (GAPDH) were identified through primer bank (https://pga.mgh.harvard.edu/primerbank/) and verified by Blast search on NCBI. Primers were synthesized by Beijing Genomics Institute (BGI). The primer sequences were as follows: claudin-11, F: 5'-CGGTGTGGCTAAGTACAGGC-3, R: 5'-CGCAGTGTAGTAGAAACGGTTTT-3'; claudin-23, F: 5'-ACGGCAGGGAGAAGACGA-3', R: 5'-AGCGACGAAGAGCACGAC-3'; GAPDH, F: 5'-CGAACTGTTTCACCAGCAAC-3'; R: 5'-GGTACATCTGGGGAACTTCT-3'.

### Protein extraction and western blot analysis

Total tissue proteins were extracted with RIPA buffer (Aidlab Biotechnologies, Beijing, China). Tissue lysates were centrifuged at 14,000 rpm for 20 min at 4°C. Supernatant was collected and protein was quantified with a BCA reagent kit. Lysates were boiled at 100°C for 5 min and 40 g of total protein was separated by 4–12% SDS-PAGE at 110 V for approximately 2 h and transferred to polyvinylidene difluoride (PVDF) membranes at 70 V for 110 min. The PVDF membranes were blocked with 5% nonfat dry milk in PBST for 1 h on a table concentrator and then incubated with rabbit polyclonal anti—claudin-11 antibody (TA334203, 1:500, Origene, Rockville, USA), rabbit polyclonal anti—claudin-23 antibody (TA334206, 1:500, Origene, Rockville, USA), and rabbit polyclonal anti- GAPDH antibody (AP0066, 1:10000, Bioworld, Minnesota, USA) in 2.5% nonfat dry milk in PBST at 4°C overnight. The membranes were then incubated with secondary anti-IgG antibody (Zb2301, 1:10000, ZSGB-Bio, Beijing, China) for 1–2 h at room temperature. Chemiluminescence reagent ECL Plus (Thermo Fisher Scientific, Massachusetts, USA) was used to visualize the bands and the results were analyzed by Image J software.

### Immunohistochemical staining

Tissues were sectioned at 4-micron thickness and mounted on positive-charged glass slides. Briefly, slides were deparaffinized in xylene, rehydrated in a graded alcohol series, and washed in tap water. The tissue sections were separately incubated in boiling sodium citrate buffer or EDTA in a steam pressure cooker for antigen retrieval. Next, endogenous peroxidase was blocked using 3% hydrogen peroxide for 10 min, and the sections were washed with phosphate-buffered saline (PBS), pH 7.4. Tissue collagen was blocked by the addition of 10% normal goat serum at 37°C for 30 min to prevent nonspecific binding. The sections were incubated with primary antibodies against claudin-11 (BS6986, 1:400, Bioworld, Minnesota, USA) and claudin-23 (TA334206, 1:500, Origene, Rockville, USA) at 37°C for 1 h. After rinsing three times with PBS for 5 min each, the sections were incubated with biotinylated secondary antibody goat anti-rabbit antibody (Maixin Inc., Fujian, China) and streptavidin-biotin peroxidase for 10 min each, followed by incubation with diaminobenzidine (DAB) as the chromogen for 1 min. Finally, the slides were rinsed with water, counterstained with hematoxylin, blued in water, dehydrated through graded alcohols, cleared in xylene, and mounted.

### Evaluation of immunohistochemical staining

Staining of claudin-11, -23 is mainly located in the cytomembrane and cytoplasm. Staining results were evaluated independently by two pathologists who were blinded to the clinicopathologic characteristics of the patients. A semiquantitative scoring criterion was used for evaluation of expression according to the intensity (0, no staining; 1, mild brown staining; 2, moderate brown staining; 3, strong brown staining) and extent (0, ≤5%; 1, 5–25%; 2, 25–50%; 3, 50–75%; 4, ≥75%) of the staining. Finally, the staining intensity and extent scores were multiplied to generate an immunoreactivity score (IS) for each specimen, which was classified as no staining, 0 point; mild staining, 1–4 points; moderate staining, 5–8 points; or severe staining, 9–12 points. A score of 0 indicated negative expression, and all other scores indicated positive expression.

### *H*. *pylori* serology examination

A 5 mL fasting venous blood sample was obtained on first admission before any adjuvant or metastatic treatment was given. All samples were centrifuged immediately at 3500 g for 10 minutes, and a serum aliquot was immediately frozen and stored until analysis. Serum anti-*Helicobacter pylori* IgG was measured using enzyme-linked immunosorbent assays (BIOHIT Plc, Helsinki, Finland) according to the manufacturer’s protocols. Duplicate negative and positive controls were included in each 96-well plate. Samples that yielded implausible values were retested. A numerical reading exceeding 34 enzyme immune units was considered to be *H*. *pylori* infection positive.

### Statistical analysis

All statistical analyses were performed using SPSS18.0 software (Chicago, IL, USA). Paired chi-square test and paired samples t-test were used to analyze differences in expression of claudin-11, -23 between gastric cancer and adjacent non-tumor tissues. Chi-square was used to analyze differences in expression of claudin-11, -23 between different gastric diseases from different individuals. The correlation between claudin-11, -23 and clinicopathologic parameters was analyzed by chi-square test and logistic multiple regression. Survival curves were estimated using the Kaplan—Meier method, and the log-rank test was used to compare differences between the curves. Multiple survival analysis was performed by Cox regression. Two-tailed P values<0.05 were considered statistically significant.

## Results

### Expression of claudin-11, -23 in gastric cancer and adjacent non-tumor tissues

**Fig 1:**
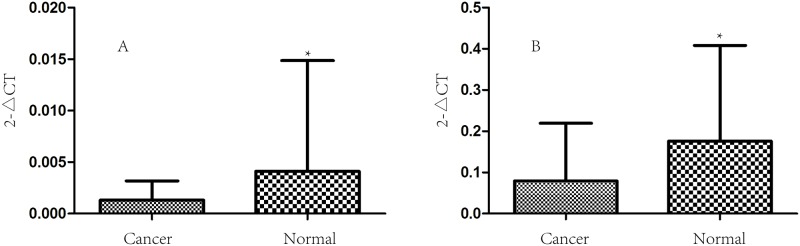
The mRNA expression of Claudin-11,23 in GC tissues and paired normal mucosa. The mRNA expression of Claudin-11,23 in 58 cases of GC tissues and paired normal mucosa. The expression status of claudin-11(A) and 23(B) in gastric cancer tissue and its adjacent normal gastric tissue were measured by real-time PCR. The results of real-time PCR showed that the expression levels of claudin-11, 23 were greatly increased in paracancerous normal gastric tissue than in cancerous tissue (0.043, 4.72*10^**−4**^).

**Fig 2:**
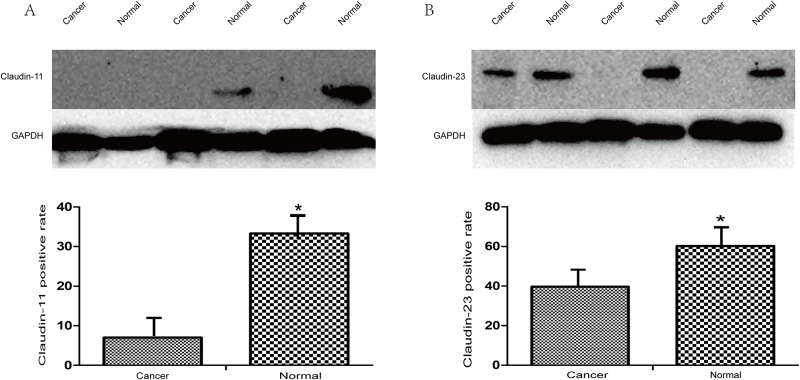
The protein expression of Claudin-11,23 in GC tissues and paired normal mucosa. The protein expression of Claudin-11,23 in 58 cases of GC tissues and paired normal mucosa. The expression level of claudin-11(A) and 23(B) in gastric cancer tissue and its adjacent normal gastric tissue were measured by western blot. The results of western blot indicated that the expression levels of claudin-11, 23 were obviously higher in paracancerous normal gastric tissue than in cancerous tissue (2.75*10^**−4**^,** 0.012**).

Real-time PCR analysis to detect the expression of claudin-11, -23 in 58 cases of gastric cancer and paracancerous superficial gastritis at the mRNA level showed that the expression of both claudin-11, -23 in gastric cancer was significantly lower than that in the paracancerous superficial gastritis (P = 0.043, P = 4.72*10^−4^ respectively; Fig 1). We also performed western blotting to detect the expression of claudin-11, -23 in 58 cases of gastric cancer and paracancerous superficial gastritis at the protein level, and found that levels of claudin-11, -23 proteins were also significantly lower in gastric cancer than that in the paracancerous superficial gastritis (P = 2.75*10^−4^, P = 0.012 respectively; Fig 2). We further used immunohistochemistry to compare the expression of claudin-11, -23 between gastric cancer and adjacent non-tumor tissues, including superficial gastritis and atrophic gastritis. The results suggested that the expression of claudin-11 in superficial gastritis was higher than that in atrophic gastritis (P = 7.63*10^−6^), and the expression in atrophic gastritis was significantly higher than that in gastric cancer (P = 9.85*10^−5^). For claudin-23, higher expression was observed in superficial gastritis than that in atrophic gastritis (P = 3.64*10^−12^) and gastric cancer (P = 9.06*10^−14^). There was no obvious difference in expression between atrophic gastritis and gastric cancer (P = 0.644) (Table 2). To explore the consistency of different detection methods, comparison was conducted between western blotting and immunochemistry staining for measuring expression levels. The result was consistent between WB and IHC in cancer tissues for claudin-23 expression (kappa = 0.854, P = 1.12*10^−10^) and marginally consistent for claudin-11 expression (kappa = 0.203, P = 0.011). Regardless of the detection method used, the general expression trend of claudin-11 and 23 was quite consistent between cancer tissues and adjacent tissues (S1 Table).

**Table 2:** Claudin-11,23 expression in SG and AG of adjacent tissue and GC.

**Claudin-11 expression**														
	**Adjacent SG**					**Adjacent AG**					**Adjacent SG**			
		**+**	**-**				**+**	**-**				**+**	**-**	
**GC**	**+**	53	1	54	**GC**	**+**	37	10	47	**Adjacent AG**	**+**	74	0	74
	**-**	55	0	55		**-**	37	9	46		**-**	18	1	19
		108	1	109			74	19	93			92	1	93
***P***	1.58*10^**−15**^				***P***	9.85*10^**−5**^				***P***	7.63*10^**−6**^			
**Claudin-23 expression**														
	**Adjacent SG**					**Adjacent AG**					**Adjacent SG**			
		**+**	**-**				**+**	**-**				**+**	**-**	
**GC**	**+**	56	1	57	**GC**	**+**	28	19	47	**Adjacent AG**	**+**	51	0	51
	**-**	49	3	52		**-**	23	23	46		**-**	39	3	42
		105	4	109			51	42	93			90	3	93
***P***	9.06*10^**−14**^				***P***	0.644				***P***	3.64*10^**−12**^			

GC, gastric cancer. AG, atrophic gastritis. SG, superficial gastritis.

### Differences in expression of claudin-11, -23 among different patients with gastric disease

**Fig 3:**
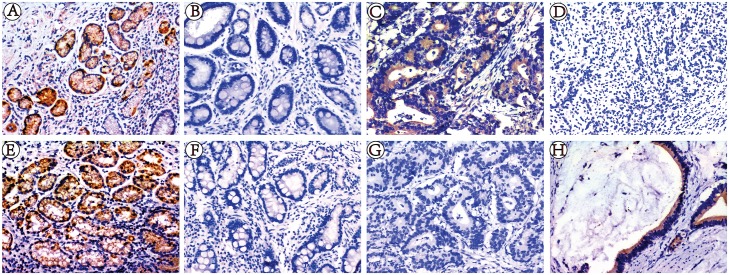
Immunohistochemical staining for Claudin-11,23 expression in SG, AG, GC. Immunohistochemical staining for Claudin-11,23 expression in 109 cases of SG, 109 cases of AG and 109 cases of GC. The staining of claudin-11, 23 are mainly located in the cytomembraneand cytoplasm. (A) SG with positive Claudin-11 expression; (B) AG with negative Claudin-11 expression;(C) moderately differentiated GC with positive Claudin-11 expression; (D) poorly differentiated GC with negative Claudin-11 expression; (E) SG with positive Claudin-23 expression; (F) AG with negative Claudin-23 expression; (G) moderately differentiated GC with negative Claudin-23 expression;(H) poorly differentiated GC with negative Claudin-23 expression. Magnification, ×200.

We also used an immunohistochemical method to compare expression of claudin-11, -23 between non-cancer individuals with superficial gastritis, atrophic gastritis, and gastric cancer. Our results showed that expression of claudin-11 in superficial gastritis was higher than that in atrophic gastritis and gastric cancer (P = 1.10*10^−9^), but there was no obvious difference between atrophic gastritis and gastric cancer (P = 0.076). The expression of claudin-23 in superficial gastritis was higher than that in atrophic gastritis and gastric cancer (P = 1.04*10^−21^), but was lower in atrophic gastritis than that in gastric cancer (P = 2.98*10^−5^) (Table 3, Fig 3).

**Table 3:** Claudin-11,23 expression in SG, AG, GC tissues from different individuals.

Group	Group1		Group2		Group3	
	SG	GC	SG	AG	AG	GC
**Claudin-11 expression**						
**Positive (%)**	104(95.4)	54(49.5)	104(95.4)	67(61.5)	67(61.5)	54(49.5)
**Negative (%)**	5(4.6)	55(50.5)	5(4.6)	42(38.5)	42(38.5)	55(50.5)
***P***	3.40*10^**−14**^		1.10*10^**−9**^		0.076	
**Claudin-23 expression**						
**Positive (%)**	97(89.0)	57(52.3)	97(89.0)	27(24.8)	27(24.8)	57(52.3)
**Negative (%)**	12(11.0)	52(47.7)	12(11.0)	82(75.2)	82(75.2)	52(47.7)
***P***	2.70*10^**−9**^		1.04*10^**−21**^		2.98*10^**−5**^	

GC, gastric cancer. AG, atrophic gastritis. SG, superficial gastritis.

In addition, we compared the expression of claudin-11, -23 between atrophic gastritis and paracancerous atrophic gastritis. The result showed that the expression of claudin-11, -23 in atrophic gastritis was significantly lower than that in paracancerous atrophic gastritis (P = 0.005, P = 1.21*10^−5^ respectively) (S2 Table).

### Correlation of claudin-11, -23 expression and clinicopathologic parameters as well as clinical biomarkers

We analyzed the associations between claudin-11, -23 expression and clinicopathologic parameters in gastric cancer. Chi-square test result showed that expression of claudin-11 in male patients was higher than that in females (P = 0.017). Patients with *H*. *pylori* infection had higher claudin-11 positivity than those without *H*. *pylori* infection (P = 0.014). High claudin-11-positive expression also significantly correlated with Borrmann grade I-II (P = 0.028). Expression of claudin-23 in patients with TNM stage I-II was obviously lower than that in patients with stage III-IV (P = 0.046), and was lower in patients with early-stage disease than that in those with progressive stage disease (P = 0.023). The expression of claudin-23 was higher in patients with vessel cancer embolus (P = 0.001) (Table 4). Further logistic multiple regression analysis indicated that claudin-11 was significantly associated with sex, smoking, alcohol, *H*. *pylori* infection and Borrmann classification while claudin-23 was only significantly associated with vessel cancer embolus (Table 4). Besides, the relationships between claudin-11 and 23 expression and clinical biomarkers such as CEA and GSTP-1 were also analyzed, and the results showed that claudin-11-positive expression significantly correlated with higher CEA expression (P = 0.039), while claudin-23 positive expression significantly correlated with GSTP1-positive expression (P = 0.048) (Table 5).

**Table 4:** Association between Claudin-11,23 expression and clinicopathological parameters in GC.

Variability	Cases(n)	claudin-11 expression		PR(%)	*P*_1_	*P*_2_	claudin-23 expression		PR(%)	*P*_1_	*P*_2_
		negative	positive				negative	positive			
**Age (years)**											
<60	51	28	23	45.1	0.384	0.385	26	25	49	0.521	0.521
≥60	58	27	31	53.4			26	32	55.2		
**Sex**											
Male	73	31	42	57.5	**0.02**	**0.02**	35	38	52.1	0.943	0.943
Female	36	24	12	33.3			17	19	52.8		
**Smoking**											
Yes	36	21	15	41.7	0.306	**0.02**	18	18	50	0.828	0.748
No	67	32	35	52.2			32	35	52.2		
**Alcohol**											
Yes	18	13	5	27.8	0.052	**0.01**	8	10	55.6	0.702	0.734
No	85	40	45	52.9			42	43	50.6		
***H*. *pylori* IgG**											
Seronegative	29	20	9	31	**0.01**	**0.02**	11	18	62.1	0.12	0.123
Seropositive	67	28	39	58.2			37	30	44.8		
**Phase of progression**											
EGC	18	11	7	38.9	0.323	0.306	13	5	27.8	**0.02**	0.175
AGC	91	44	47	51.6			39	52	57.1		
**Borrmann classification**											
Borrmann I—II	24	7	17	70.8	**0.03**	**0.05**	12	12	50	0.41	0.915
Borrmann III—IV	67	37	30	44.8			27	40	59.7		
**Lauren’s classification**											
Intestinal-type	24	11	13	54.2	0.535	0.715	11	13	54.2	0.92	0.485
Diffuse-type	83	44	39	47			39	44	53		
**TNM stage**											
I—II	52	26	26	50	0.927	0.861	30	22	42.3	**0.05**	0.173
III—IV	57	29	28	49.1			22	35	61.4		
**Lymph node metastasis**											
Positive	62	32	30	48.4	0.782	0.512	25	37	59.7	0.076	0.08
Negative	47	23	24	51.1			27	20	42.6		
**T stage**											
T1	18	12	6	33.3	0.276	0.41	12	6	33.3	0.374	0.575
T2	16	6	10	62.5			7	9	56.3		
T3	11	4	7	63.6			5	6	54.5		
T4	64	33	31	48.4			28	36	56.3		
**Vessel cancer embolus**											
Positive	53	27	26	49.1	0.922	0.791	17	36	67.9	**0**	**0.01**
Negative	56	28	28	50			35	21	37.5		
**Perineural invasion**											
Positive	74	38	36	48.6	0.657	0.741	33	41	55.4	0.258	0.209
Negative	28	13	15	53.5			16	12	42.9		
**Tumor size**											
≤3	62	31	31	50	0.858	0.809	28	34	54.8	0.158	0.394
3–5	33	16	17	51.5			14	19	57.6		
≥5	14	8	6	42.9			10	4	28.6		
**Family history**											
Yes	79	43	36	45.6	0.196	0.2	40	39	49.4	0.546	0.546
No	23	9	14	60.9			10	13	56.5		

GC, gastric cancer. EGC, early stage of gastric cancer. AGC, advanced stage of gastric cancer. PR, Positive rate. ref, reference. *P*_1_, results of chi-square test. *P*_2_, results of logistic multiple regression.

**Table 5:** Association between Claudin-11,23 expression and CEA and GSTP1.

Variability	Cases(n)	claudin-11 expression		PR(%)	*P*	claudin-23 expression		PR(%)	*P*
		negative	positive			negative	positive		
**CEA**									
Positive	64	27	37	57.8	**0.039**	31	33	51.6	0.855
Negative	45	28	17	37.8		21	24	53.3	
**GSTP1**									
Positive	59	27	32	54.2	0.287	23	36	61	**0.048**
Negative	50	28	22	44		29	21	42	

### Relationship between the expression of claudin-11, -23 and the prognosis of gastric cancer

**Fig 4:**
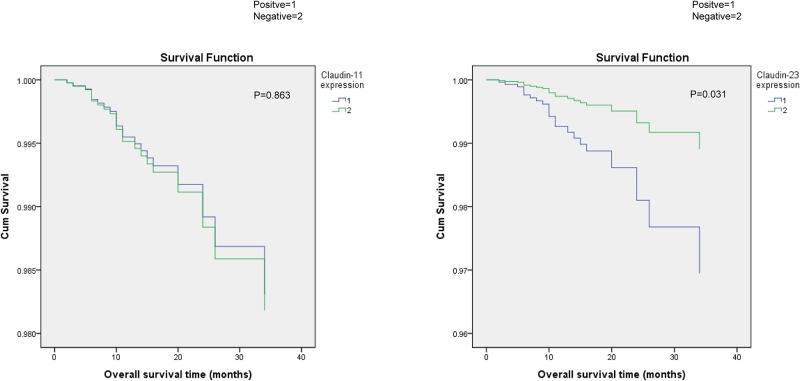
Correlation of Claudin-11,23 expression with survival curves of gastric cancer patients by univariate survival analysis. Correlation of Claudin-11, 23 expression with survival curves of gastric cancer patients by univariate survival analysis. (A) Kaplan-Meier survival curves comparing months of survival in gastric cancer patients are shown for Claudin-11 expression (P = 0.863); (B) Kaplan-Meier survival curves comparing months of survival in gastric cancer patients are shown for Claudin-23 expression (P = 0.031).

According to univariate survival analysis, we found no significant correlation between the expression of claudin-11 and the prognosis of gastric cancer (P = 0.594), whereas the expression of claudin-23 was significantly related to poor prognosis (P = 0.01). Besides, age (P = 0.039), TNM stage (P = 2.37*10^−4^), lymph node metastasis (P = 0.001), vessel cancer embolus (P = 0.007), and perineural invasion (P = 0.043) were all closely related to the prognosis of gastric cancer (S3 Table). Because TNM stage already comprised information on lymph node metastasis, we performed multivariate analysis using Cox’s proportional hazards model adjusted by age, TNM stage, vessel cancer embolus, perineural invasion. The results indicated that the survival time decreased along with the expression intensity of claudin-23 increased, which implied that claudin-23 positivity was an independent prognostic factor (P = 0.031, hazard ratio = 2.826, 95% confidence interval 1.098–7.270), and that patients with positive expression had a shorter survival (Fig 4, Table 6, S4 Table).

**Table 6:** Correlation between Claudin-11,23 expression and survival in GC.

	Case	Cases of Events	MST	Univariate			Multivariate		
				*P*	HR	95%CI	*P*	HR	95%CI
**Claudin-11 expression**									
Positive	54	11	34.5		1(ref)			1(ref)	
Negative	55	14	32.1	0.594	0.806	0.366–1.778	0.863	0.930	0.406–2.128
**Claudin-23 expression**									
Positive	57	18	28.7		1(ref)			1(ref)	
Negative	52	7	38.1	**0.01**	3.175	1.318–7.645	**0.031**	2.826	1.098–7.27

GC, gastric cancer. MST, median survival time; HR: hazard radio; CI: confidence interval. ref, reference.

## Discussion

Claudin proteins are critical for tight junction function, and their up/downregulation can disrupt the structure and function of tight junctions, leading to the loss of intracellular adhesion and loss of homeostasis [22]. In addition, abnormal expression of claudin proteins can affect cell proliferation, differentiation, apoptosis, and invasion through many signaling pathways that play important roles in the progression of cancer [23]. In this study, we focused on two major members of the claudin family, claudin-11, -23, and examined their expression in gastric cancer, atrophic gastritis, and superficial gastritis. Moreover, we investigated the relationships between expression of claudin-11, -23 and clinicopathologic parameters and survival in gastric cancer.

Previous studies reported that claudin-11, -23 are abnormally expressed in many malignant tumors. For instance, claudin-11 expression was higher in normal cholecyst tissues than that in cholecyst carcinoma tissues [12], whereas claudin-23 expression was downregulated in colonic carcinoma [14]. However, the expression of claudin-11, -23 in gastric cancer has been rarely reported and the few results are controversial; some studies showed that claudin-11 expression was upregulated in gastric cancer [16]whereas Agarwal et al. came to the opposite conclusion [17]. Katoh et al. [18]found that claudin-23 mRNA expression was downregulated in intestinal type of gastric cancer, but its expression in gastric cancer at the protein level was unclear. In this study, we used real-time PCR, western blotting, and immunohistochemical methods to detect the expression of claudin-11, -23 in different gastric diseases, including superficial gastritis, atrophic gastritis, gastric cancer, and adjacent non-tumor tissues. Our results showed that the expression of claudin-11 was lower in gastric cancer than that in superficial gastritis, which is in accordance with the results of Agarwal et al. [17]. The expression of claudin-23 was also lower in gastric cancer than that in superficial gastritis at both the mRNA and protein level, which further confirmed previous reports of downregulation of claudin-23 mRNA in intestinal gastric cancer [18]. The underlying mechanisms of the abnormal expression of claudin-11, -23 in gastric cancer remain unclear. It is widely accepted that multiple mechanisms may participate in the regulation of claudin expression in gastric cancer. For instance, the transcription factor Cdx2 can enhance the expression of claudin-3 and -4 in gastric cancer cells [24], and the transcription factor RUNX3 can enhance the expression of claudin-1 by binding to its promoter region [25]. In addition, epigenetic mechanisms also play important roles in regulating the expression of claudin proteins. The expression of claudin-4 in gastric cancer was negatively associated with DNA methylation [26], and miRNA can lead to decreased expression of claudin-18 by binding to its 3'-UTR region and subsequently promote the proliferation, migration, and invasion of gastric cancer cells [27]. Furthermore, post-translational modification of the C-terminus of claudin protein, such as phosphorylation, is another important regulatory mechanism for the expression of claudin proteins. Phosphorylation of claudin-5 induced by cyclic adenosine monophosphate and phosphorylation of claudin-1 by protein kinase C can both destroy tight junction barrier function [28, 29]. Further investigation into the mechanism underlying the abnormal expression of claudin-11, -23 in gastric cancers is necessary.

In addition, as an important precancerous disease, we found that the expression of claudin-11, -23 was also significantly lower in atrophic gastritis than superficial gastritis. Moreover, we found an interesting phenomenon that the expression of claudin-11, -23 in atrophic gastritis was significantly lower than that in paracancerous atrophic gastritis. Simple atrophic gastritis tissues come from non-cancer patients, while the paracancerous atrophic gastritis tissues come from patients with gastric cancer. Although both of them have the morphological changes of atrophic features, the two kinds of atrophic gastritis can be significantly different in terms of biological behavior. It has been reported that the biological characteristics of paracancerous mucosa have been changed significantly. For example, Liu GS revealed that the proportion of type III intestinal metaplasia (incomplete type of intestinal metaplasia) was significantly higher in intestinal metaplasia foci in adjacent noncancerous atrophic gastritis than in atrophic gastritis with intestinal metaplasia [30]. Besides, Gonzalez, CA found that incomplete type of intestinal metaplasia has the highest risk to progress to gastric cancer [31]. All the above may lead to the different expression of claudin-11, -23 between atrophic gastritis and paracancerous atrophic gastritis. However, further study is necessary to explore the biological characteristics of the paracancerous lesions.

We also investigated the relationships between claudin-11, -23 and clinicopathologic parameters. Our results showed that expression of claudin-11 was significantly correlated with sex, smoking, alcohol, *H*. *pylori* infection and Borrmann classification, whereas expression of claudin-23 was correlated with vessel cancer embolus. These results indicated that expression of claudin-11, -23 is associated with a number of clinicopathologic parameters reflecting gastric cancer development, and might thus play important roles in predicting the biologic activities and progression of gastric cancer. In addition, the expression of claudin-11 in gastric cancer with *H*. *pylori* infection was significantly higher than that in that without *H*. *pylori* infection (58.3% vs. 81.3%, P = 0.014), indicating that infection with *H*. *pylori* might affect the expression of claudin-11. *H*. *pylori* infection is one of the main causes of gastric carcinogenesis, and it has previously been reported that *H*. *pylori* infection can upregulate the expression of claudin proteins [32]. A better understanding of the mechanism of claudin-11 upregulation induced by *H*. *pylori* infection might provide effective therapeutic targets for diseases related to *H*. *pylori* infection.

We further investigated the relationships between claudin-11, -23 expression and the prognosis of gastric cancer patients. According to univariate and multivariate survival analyses, the results showed that decreased claudin-23 expression had association with survival whereas much lower expression of claudin-11 had no association with survival. This non-linear relationship between claudin expression and cancer prognosis has been reported before. For example, Wang et al. found that low claudin-6 expression indicate a worse prognosis in patients with non-small cell lung cancer [33] and Li et al. showed that high expression of claudin-1 had a poorer prognosis than those with low expression in patients with hypopharyngeal squamous cell carcinoma [34]. In this regard, we speculated that whether claudin expression affects cancer prognosis is largely dependent on their basic function instead of differential expression level. Although claudin-11, -23 both belong to claudin family, these two proteins have different functions due to the different in C-terminal structure, which included 25–55 amino acids for claudin-11 and 111 amino acids for claudin-23. C-terminal is the most important structure which can affect claudin protein’s physiological function, such as regulating cell proliferation, apoptosis, migration and invasion via related signal pathway[35–37]. Moreover, we found that claudin-11, -23 had different correlation with CEA and GSTP-1 expression. The results showed that claudin-11 positive expression was significantly correlated with higher expression of CEA, a commonly used indicator for clinical diagnosis of gastric cancer [38]; while claudin-23 positive expression was significantly associated with positive expression of GSTP1, which is closely related with chemotherapy insensitivity and may lead to poor prognosis [39]. This may further explain why claudin-23 was more associated with survival. Nevertheless, our data suggest that claudin-23 expression may have potential prognostic value in gastric cancer, and the underlying mechanisms should be clarified by further studies.

In summary, downregulation of claudin-11, -23 in gastric cancer correlated with some clinicopathologic features; in particular, claudin-23 showed potential for development as a prognosticator of patient survival. Our results showed that claudin-11, -23 were closely related to the occurrence and progression of gastric cancer, and may therefore serve as potential biomarkers for the diagnosis and prognosis of gastric cancer.

## Supporting information

S1 Table Comparisons of different methods for measuring expression levels. (DOCX)

S2 Table Different expression of claudin-11, 23 between AG and adjacent AG. (DOCX)

S3 Table Clinicopathological parameters and survival in GC. (DOCX)

S4 Table The survival time of patients with different claudin-23 expression. (DOCX)
