# Supplementary material for: Expression of claudin-11, -23 in different gastric tissues and its relationship with the risk and prognosis of gastric cancer
Source: PLoS One. 2017 Mar 28;12(3):e0174476. doi: 10.1371/journal.pone.0174476 (PMC5369768; doi:10.1371/journal.pone.0174476)
Supplement: S1 Table — (DOCX) [file pone.0174476.s001.docx]

| **S1 table. Comparisons of different methods for measuring expression levels.** | | | | | | | | | | |
| --- | --- | --- | --- | --- | --- | --- | --- | --- | --- | --- |
|  | **Claudin-11 expression** | | | | | | | | | |
|  | **Normal tissues** | | | |  | **Cancer tissues** | | | | |
|  | **Immunochemistry staining** | | | |  |  | **Immunochemistry staining** | | | |
|  |  | **+** | **-** |  |  |  |  | **+** | **-** |  |
| **Western blot** | **+** | 19 | 0 | 19 |  | **Western blot** | **+** | 4 | 0 | 4 |
|  | **-** | 39 | 0 | 39 |  |  | **-** | 19 | 35 | 54 |
|  |  | 58 | 0 | 58 |  |  |  | 23 | 35 | 58 |
| ***Kappa*** | **/** | | | |  | ***Kappa*** | **0.203** | | | |
| ***P*** | **/** | | | |  | ***P*** | **0.011** | | | |
|  | **Claudin-23 expression** | | | | | | | | | |
|  | **Normal tissues** | | | |  | **Cancer tissues** | | | | |
|  | **Immunochemistry staining** | | | |  |  | **Immunochemistry staining** | | | |
|  |  | **+** | **-** |  |  |  |  | **+** | **-** |  |
| **Western blot** | **+** | 35 | 0 | 35 |  | **Western blot** | **+** | 21 | 2 | 23 |
|  | **-** | 23 | 0 | 23 |  |  | **-** | 3 | 32 | 35 |
|  |  | 58 | 0 | 58 |  |  |  | 24 | 34 | 58 |
| ***Kappa*** | **/** | | | |  | ***Kappa*** | **0.854** | | | |
| ***P*** | **/** | | | |  | ***P*** | **1.12*10^-10^** | | | |
