# Supplementary material for: Expression of claudin-11, -23 in different gastric tissues and its relationship with the risk and prognosis of gastric cancer
Source: PLoS One. 2017 Mar 28;12(3):e0174476. doi: 10.1371/journal.pone.0174476 (PMC5369768; doi:10.1371/journal.pone.0174476)
Supplement: S2 Table — (DOCX) [file pone.0174476.s002.docx]

| **S2 Table. Different expression of claudin-11, 23 between AG and adjacent AG** | | | | | | | |
| --- | --- | --- | --- | --- | --- | --- | --- |
| **Variability** | **AG** | | **PR(%)** | **Adjacent AG** | | **PR(%)** | ***P*** |
|  | **positive** | **negative** |  | **positive** | **negative** |  |  |
| **claudin-11 expression** | 67 | 42 | 61.5 | 74 | 19 | 79.6 | **0.005** |
| **claudin-23 expression** | 27 | 82 | 24.8 | 51 | 42 | 54.8 | **1.21*10^-5^** |
| AG, atrophic gastritis. | | | | | | | |
