# Supplementary material for: Expression of claudin-11, -23 in different gastric tissues and its relationship with the risk and prognosis of gastric cancer
Source: PLoS One. 2017 Mar 28;12(3):e0174476. doi: 10.1371/journal.pone.0174476 (PMC5369768; doi:10.1371/journal.pone.0174476)
Supplement: S3 Table — (DOCX) [file pone.0174476.s003.docx]

| **S3 Table. Clinicopathological parameters and survival in GC.** | | | |
| --- | --- | --- | --- |
| **Variable** | **Cases of Events** | **MST** | ***P*** |
|  |  |  |  |
| **Age (years)** |  | | |
| <60 | 7 | 37.683 | **0.039** |
| ≥60 | 18 | 29.323 |  |
| **Sex** |  |  | 0.166 |
| Male | 14 | 35.078 |  |
| Female | 11 | 30.505 |  |
| **Smoking** |  |  | 0.104 |
| Yes | 5 | 37.913 |  |
| No | 19 | 31.334 |  |
| **Alcohol** |  |  | 0.180 |
| Yes | 2 | 38.086 |  |
| No | 22 | 32.03 |  |
|  |  |  |  |
| ***H. pylori* IgG** |  |  | 0.908 |
| Seronegative | 8 | 34.627 |  |
| Seropositive | 14 | 32.655 |  |
| **Phase of progression** |  |  | 0.337 |
| EGC | 0 | / |  |
| Borrmann I–II | 4 | 33.858 |  |
| Borrmann III–IV | 21 | 29.583 |  |
| **Lauren’s classification** |  |  | 0.143 |
| Intestinal-type | 2 | 37.183 |  |
| Diffuse-type | 23 | 31.742 |  |
| **TNM stage** |  |  | **2.37*10^-4^** |
| I–II | 2 | 42.452 |  |
| III–IV | 23 | 24.914 |  |
| **Lymph node metastasis** |  |  | **0.001** |
| Positive | 22 | 26.179 |  |
| Negative | 3 | 41.494 |  |
| **T stage** |  |  | 0.103 |
| T1 | 0 | / |  |
| T2 | 0 | / |  |
| T3 | 0 | / |  |
| T4 | 25 | 26.243 |  |
| **Vascular invasion** |  |  | **0.007** |
| Positive | 18 | 25.972 |  |
| Negative | 7 | 37.903 |  |
| **Perineural invasion** |  |  | **0.043** |
| Positive | 24 | 25.972 |  |
| Negative | 0 | 37.903 |  |
| **Tumor size** |  |  |  |
| ≤3 | 0 | / |  |
| 3-5 | 5 | 37.549 |  |
| >5 | 20 | 26.105 |  |
| **Family history** |  |  | 0.185 |
| Yes | 8 | 28.049 |  |
| No | 16 | 34.566 |  |
| GC, gastric cancer;  MST, median survival time. | | | |
