# Supplementary material for: Expression of claudin-11, -23 in different gastric tissues and its relationship with the risk and prognosis of gastric cancer
Source: PLoS One. 2017 Mar 28;12(3):e0174476. doi: 10.1371/journal.pone.0174476 (PMC5369768; doi:10.1371/journal.pone.0174476)
Supplement: S4 Table — (DOCX) [file pone.0174476.s004.docx]

| **S4 Table. The survival time of patients with different Claudin-23 expression** | | | |
| --- | --- | --- | --- |
|  | **Claudin-23 expression** | | |
|  | **-** | **+** | **++** |
| **MST** | 38.071 | 28.056 | 18.6 |
| MST, median survival time. | | | |
